# Effects of Pomegranate Peel Extract and/or Lactic Acid as Natural Preservatives on Physicochemical, Microbiological Properties, Antioxidant Activity, and Storage Stability of Khalal Barhi Date Fruits

**DOI:** 10.3390/foods12061160

**Published:** 2023-03-09

**Authors:** Nashi K. Alqahtani, Tareq M. Alnemr, Salim A. Ali

**Affiliations:** 1 Department of Food and Nutrition Sciences, College of Agricultural and Food Sciences, King Faisal University, Al-Ahsa 31982, Saudi Arabia; talnemr@kfu.edu.sa; 2 Department of Food Science, Faculty of Agriculture (Saba Basha), Alexandria University, Alexandria 21531, Egypt

**Keywords:** Barhi dates, pomegranate peel extract, cold storage, antioxidant activity, sensory characteristics, microbiological quality

## Abstract

The use of natural preservatives in the storage of fresh fruits is a promising approach to healthier and more sustainable food processing. The current study was conducted to evaluate the effect of pomegranate peel extract (PPE) and lactic acid (LA) as natural preservatives on the shelf life of Khalal Barhi date fruits. Physicochemical properties, antioxidant activity, color parameters, texture profile, sensory characteristics, and microbiological quality of date fruits were evaluated during six weeks of cold storage (4 ± 1 °C). The bioactive compounds in PPE were determined by HPLC analysis, which showed that PPE is a rich source of bioactive compounds, particularly phenolics and flavonoids. The results exhibited decreased moisture content (from 68.36–68.43 to 62.13–63.11%) and an increased soluble solids content (from 27.16–27.24 to 31.45–33.91%) in all samples with prolonged storage. Likewise, a slight decrease in the pH (from 6.00–6.28 to 4.89–5.29) with an increase in acidity (from 0.12–0.14 to 0.23–0.27%) during storage was observed. Generally, treated samples showed lower changes in moisture content, soluble solids content, pH, and acidity than the control. A decrease in total phenolic content (TPC) (from 8.22–12.36 to 3.19–5.17 mg GAE/g) and antioxidant activity (from 44.11–68.26 to 23.84–38.52%) of all samples was observed with progressed storage, meanwhile, the treatment with PPE significantly (*p* < 0.05) increased TPC with a concomitant increase in antioxidant activity and maintained higher values of both throughout storage. The results exhibited a decrease in L* (from 54.87–55.92 to 41.68–45.68) and b* (from 36.25–37.09 to 23.59–25.42) values of all samples, while the a* value of all samples increased (from 1.29–1.40 to 2.22–2.43) with storage. Dipping treatment in PPE solution improved the color, exhibited the lowest change in color parameters, and maintained better texture properties during storage. Similarly, sensory properties decreased (from 8.21–8.66 to 6.71–7.21) during storage with insignificant (*p* > 0.05) differences among samples. Dipping treatments inhibited the growth of yeasts and molds over time, with the lowest microbial count recorded in the PPE-treated samples. It can be concluded that PPE was found to have protective effects on Khalal Barhi date fruit quality by controlling post-harvest changes and lowering the microbial load.

## 1. Introduction

**Figure 1:**
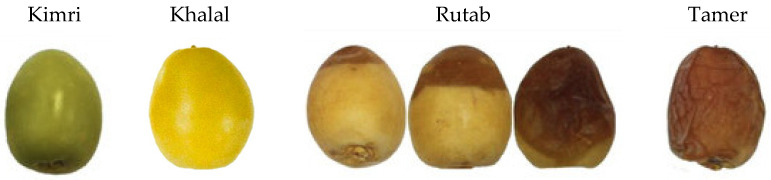
Different maturity stages of Barhi date fruits.

The date palm (*Phoenix dactylifera* L.) is one of the most important and indispensable fruit crops in many countries, especially in the Middle East [1,2]. Saudi Arabia is ranked second among top date palm-producing countries with an annual production of 1.57 million tons [3]. Numerous studies have shown that dates, in addition to their function in agroecosystems, have a high nutritional value, multiple health advantages, and many economic benefits [4]. Date palm is a climacteric crop in which the fruits are subjected to certain physiological changes during maturation due to the production of the ethylene hormone [5]. The ripening process of date fruits could be divided into four stages (Figure 1) as follows: (1) Kimri stage refers to immature fruits with hard texture and green color; (2) Khalal stage reflects hard, yellow, or red (depending on the variety), edible date fruits with a delicious taste and desirable texture; (3) Rutab stage refers to soft, brown, and semi-ripe date fruits; and (4) Tamer stage, which displays date fruits that are fully ripe, more soft, and dark brown [6].

Barhi, a popular date variety, is widely consumed at the Khalal stage of maturity. Due to its significant market and consumer relevance, distinct organoleptic qualities, and related health advantages, it is in high demand. Such favored quality characteristics could be due to the presence of bioactive compounds and flavor components. However, Barhi dates, if not preserved in a proper condition, will rapidly convert from Khalal to Rutab [7]. Hence, it is of vital importance to find appropriate methods to increase the shelf-life of Barhi dates at the Khalal stage and maintain their excellent qualities after harvest and during the marketing process for a longer period. A few methods that have been used to reduce quality changes in fruits during storage, such as chemical preservatives, controlled atmosphere storage, modified atmosphere packaging, disinfection solutions, electron beams, UV-C light, electrolyzed water, and ozone treatment [5,8].

Recently, the utilization of plant extracts as natural preservatives became a promising trend in the food industry [9]. In the last few years, upcycling agro-industrial by-products to create value-added products has received a lot of attention in the food industry [10]. Such a tendency could contribute to reducing environmental pollution, modernizing low-value products, creating new functional foods, and thereby enhancing the local and global economies in line with the UN sustainable development goals [11,12]. The primary by-product of the pomegranate (*Punica granatum* L.) juice industry is pomegranate peel, which makes up about 40–50% of the total fruit weight [13]. The pomegranate peel extract (PPE) can be used as a safe, low-cost, value-added material, and as a degradable preservative when applied to food products [14]. PPE is a rich source of bioactive compounds particularly phenolic acids (such as gallic acid, ellagic acid, p-coumaric acid, caffeic acid, chlorogenic acid, ferulic acid, syringic acid, vanillic acid, and cinnamic acid), flavonoids (e.g., anthocyanins rutinosides, pentosides and glucosides of cyanidin, pelargonidin, and delphinidin), and tannins (e.g., ellagitannins, gallotannins, punicalagin, punicalin, castalagin, corilagin, granatins, pedunculagin, and tellimagrandin) [13,15,16]. As a result, numerous researchers verified PPE’s antimicrobial and antioxidant properties [13,14,17,18]. Lactic acid (LA), generally recognized as safe (GRAS) substance, has a potent antimicrobial impact on a variety of food spoilage and pathogenic microorganisms, therefore, it could be utilized as an efficient preservative [19]. The application of LA in the processing of fruits and vegetables was also approved by the National Organic Program in the USA [20]. Moreover, previous studies confirmed the sanitizing effect of LA in various food products, i.e., apples, broccoli, red chicory, meat, and poultry products [19,20,21,22]. A previous study, conducted by Seddiek et al. [19] on cold-stored apple fruits, revealed that using LA and PPE together improved the preservation efficiency more than using PPE or LA alone. Therefore, we used LA to improve the efficiency of PPE as a natural preservative for postharvest produce.

The purpose of the current study was to find out how PPE and LA affected the qualities of fresh Barhi date fruits at the Khalal stage through monitoring the changes in physicochemical properties, phenolic content, antioxidant activity, color parameters, texture profile, sensory characteristics, and microbial load of the dates during cold storage besides the HPLC analysis of PPE.

## 2. Materials and Methods

### 2.1. Materials

Fresh Barhi date fruits (*P. dactylifera* L.), in the Khalal stage, and fresh pomegranate fruits (*P. granatum* L.) were obtained from a private farm in Al-Hofuf, Al-Ahsa, Saudi Arabia. The experiment used date fruits that had not been physically harmed, free of microbial spoilage, and had not been bitten by insects. Chemicals and reagents (NaOH, phenolphthalein, Folin–Ciocalteu reagent, gallic acid, sodium carbonate, DPPH solution) were procured from Sigma-Aldrich Co., Steinheim, Germany. Storage packages of low-density polyethylene were purchased from the local market.

### 2.2. Preparation of Pomegranate Peel Extract (PPE)

The pomegranate fruits were washed with tap water and peeled manually; the peels were dried at 40 °C in an electric tray dryer for two days. Then, the dried peels (moisture 7.21%) were ground with a kitchen grinder to pass through a 60-mesh sieve and stored at 4 °C until extraction. Pomegranate peel powder (50 g) was mixed with 500 mL distilled water, stirred at 70 °C for 4 h, cooled at room temperature, and filtered through filter paper to remove impurities. The filtered residue was re-extracted with 100 mL of water and a substantial quantity of extract was obtained. The extract was pooled and freeze-dried using a lyophilizer (Model 4KBTXL-75, VirTis SP Scientific, PA, USA). The final dry extract was powdered and resuspended in distilled water to a final concentration of 10% (w/v) and kept in a sterilized dark bottle at 4 °C until use [7,14].

### 2.3. HPLC Analysis of Polyphenolic Compounds of PPE

The phenolic and flavonoid compounds in PPE were characterized and quantified by HPLC (Model Agilent 1260 Infinity HPLC Series, Agilent Technologies, CA, USA) with an Eclipse C18 column (250 × 4.6 mm, 5 μm particle size), a quaternary pump VL, and a UV/Vis detector according to a method described earlier, with few modifications [23,24]. The separation was carried out using a mobile phase consisting of a mixture of solvent A (HPLC-grade water) and solvent B (acetonitrile containing 0.05% trifluoroacetic acid) with a flow rate of 0.9 mL/min and an isocratic elution (80:20) program. The injection volume of the sample solution was 5 μL, the column temperature was adjusted to 40 °C, the detection wavelength was set at 280 nm, and the chromatogram was developed and analyzed using the Agilent Chem Station. The following standards were procured from Merck KGaA (Darmstadt, Germany): gallic acid, chlorogenic acid, catechin, methyl gallate, caffeic acid, syringic acid, pyro catechol, rutin, ellagic acid, coumaric acid, vanillin, ferulic acid, naringenin, daidzein, quercetin, cinnamic acid, apigenin, kaempferol, and hesperetin. The bioactive compounds in the PPE were identified by comparing the information included in their UV spectra to those of the used standards. The phenolic and flavonoid content was determined by calculating the area under the peak of each compound on the chromatogram. The quantification of standards is given in Supplementary Materials (Table S1). Determinations were performed in duplicate, and the results were expressed as mg/g of PPE.

### 2.4. Experimental Design

Fresh Barhi date fruits were dipped for 5 min in the following four disinfection solutions: LA (1%), PPE (0.1%), LA (1%) + PPE (0.1%), and distilled water without any additions for the control group. The dipping solution concentration used was selected according to our previous study [19]. Then, samples were allowed to dry on a sanitized sieved stand at room temperature for 2–3 h and an electric fan was used for rapid moisture removal. Finally, all samples were packed in polyethylene plastic containers (250 g ≈ 13 date) with 7 packages for each treatment and they were stored at 4 ± 1 °C and 80% RH [25] for further determination. Samples were collected at 0, 1, 2, 3, 4, 5, and 6 weeks of storage.

### 2.5. Physicochemical Properties

All determinations were performed without washing the samples to reflect the actual properties of the treatments. The direct oven drying method was used to determine the moisture content of the date samples and dried pomegranate peels [7]. Water activity was estimated with a water activity meter (Model Aqualab CX3-TE, Labo-Scientifica, Parma, Italy). The total soluble solids (TSS) content was determined by a hand-held refractometer (Model N-50E, Brix 0–50%, ATAGO^®^, Tokyo, Japan). The pH values for the slurry prepared by blending 10 g of the sample in 20 mL of distilled water for 2 min were measured using a digital pH meter (Model AD1030, ADWA^®^, Szeged, Hungary). The titratable acidity (TA) was determined by following the method described earlier [19] using NaOH solution (0.1 N) for titration and the results were expressed as the percentage of malic acid per 100 g sample.

### 2.6. Total Phenolic Content

The total phenolic content (TPC) of date samples was estimated according to the method described by Corrêa et al. [26], using Folin–Ciocalteu’s reagent. A spectrophotometer (Model 6405 UV/VIS, JENWAY^®^, Staffordshire, UK) was used to measure the absorbance at 765 nm. The TPC was calculated in milligrams of gallic acid equivalent (GAE) per gram of material.

### 2.7. Antioxidant Activity

The DPPH radical scavenging technique, as described by Ghafoor et al. [7], was used to assess the antioxidant activity of the date samples. A spectrophotometer was used to measure the absorbance readings at 517 nm, and the percentage of DPPH inhibition was reported.

### 2.8. Color Measurement

The color properties of the date samples were evaluated before and during storage using a portable HunterLab spectrophotometer (Model MiniScan^®^ EZ 4500L, Hunter Associates Laboratory Inc., Reston, VA, USA). The device was first standardized using a black tile followed by a white tile according to the determination guidelines. The measurements were taken in three different regions on the surface and the average was calculated directly by the device. The readings were taken in triplicates for each sample. The results were presented as values of L* (0 = darkness, 100 = lightness), a* (−60 = greenness, +60 = redness) and b* (−60 = blueness, +60 = yellowness) [23,27].

### 2.9. Texture Profile Analysis

Using a texture analyzer (Model HD3128, Stable Micro Systems, Surrey, UK), the parameters of the texture profile [hardness (kg), cohesiveness, and springiness (mm)] of Barhi date samples were examined. The test was carried out using a cylindrical probe (P75) at a velocity of 1.5 mm/s and a depth of 5 mm. The measurements were taken in triplicate using a two-cycle test according to the method described by Alhamdan et al. [28]. All determinations were made at a controlled room temperature (25 °C).

### 2.10. Sensory Evaluation

The sensory properties of date samples were evaluated on the first day of treatment and then after each week of storage. Ten semi-trained panelists were recruited to perform the test. According to the procedure outlined by Kumar et al. [29] the panelists were asked to assess the samples’ appearance, odor, taste, texture, and overall acceptability using the nine-point hedonic scale.

### 2.11. Yeasts and Molds Count

Following the steps outlined by Voon et al. [30], the standard spread plate method was used to determine the total number of yeasts and molds by mixing 25 g of the samples aseptically with 225 mL of sterile saline solution in a Stomacher blender for 1 min. Then, 0.1 mL of the diluted sample was spread on a Rose-Bengal chloramphenicol agar (Oxoid CM0549). The colonies were counted after 3–5 days of incubation at 25 °C and expressed as log CFU/g.

### 2.12. Statistical Analysis

The results were presented as the mean ± standard deviation of three replicates. Two-way analysis of variance (ANOVA) was carried out to evaluate the significant differences (*p* < 0.05) between the treatments. Duncan’s multiple range test was applied to compare the means using statistical software (SPSS Inc., Chicago, IL, USA).

## 3. Results and Discussion

### 3.1. HPLC Analysis of PPE

The HPLC examination of PPE revealed that polyphenols, like phenolics and flavonoids, were present in substantial concentrations. Ellagic acid and gallic acid were the two main phenolic compounds contained in PPE, according to the HPLC results (Table 1 and Figure S1) with quantities of 35.12 and 12.50 mg/g, respectively. In addition, six phenolic compounds with lower concentrations (chlorogenic acid, methyl gallate, caffeic acid, syringic acid, vanillin, and ferulic acid) were also detected through HPLC analysis. Likewise, the results exhibited that catechin and naringenin were the two major flavonoids found in PPE with concentrations of 4.10 and 1.29 mg/g, respectively. Moreover, two flavonoids, namely rutin and apigenin, also appeared on the HPLC chromatogram of PPE with low concentrations. Our findings are in line with those obtained by Mosa et al. [31], who reported that the main polyphenols in PPE include gallic acid, ellagic acid, quercetin, caffeic acid, p-coumaric acid, and vanillic acid. Fischer et al. [32] investigated the polyphenols in pomegranate peels and they observed similar results but with different concentrations. However, it is difficult to compare the current data with those in the literature due to the differences in the extraction methods, pomegranate variety, agricultural, and environmental conditions such as soil quality and sunlight [33]. 

It is important to remember that the phenolics (mainly ellagic acid, gallic acid, chlorogenic acid, and caffeic acid) and flavonoids (particularly catechin and naringenin) found in PPE are what give it its antimicrobial and antioxidant qualities [14,34]. These compounds include hydrolysable tannins and phenolic acids, which have phenolic hydroxyl groups and double bonds, which may be responsible for the antimicrobial effect [14,23]. The HPLC findings are in line with the results of microbial count (Section 3.7), where the samples treated with LA + PPE or PPE showed lower microbial load and longer shelf life (5 and 6 weeks, respectively) compared to control and LA-treated samples (3 and 4 weeks, respectively). As is consistent with the antioxidant activity results (Section 3.3), the PPE and PPE + LA samples showed higher antioxidant activity at day zero and during the storage period compared to control and LA-treated samples, which indicates that PPE improved the antioxidant activity of Barhi date fruits.

### 3.2. Physicochemical Properties

Table 2 displays the variations in moisture content, water activity, total soluble solids (TSS), pH, and titratable acidity of fresh Barhi dates treated with natural preservatives and stored at 4 ± 1 °C. Fruits should be examined periodically during storage, as the loss of water content is a sign of quality degradation. Generally, over the storage period, the moisture content of all samples dropped (from 68.36–68.43 to 62.13–63.11%) significantly (*p* < 0.05). However, the moisture content of all samples remained above 50% up to the end of storage. The moisture loss that occurred during storage could be due to the conversion of date fruits from the Kimri to the Rutab stage [7]. In addition, Seddiek et al. [19] reported that the water loss may be attributed to the natural catabolic processes due to respiration, senescence, and other metabolic processes during storage [19]. Samples treated with natural preservatives (LA, PPE, LA + PPE) showed less moisture loss than the control. Moreover, the PPE treatment was more effective in preventing moisture loss than other treatments. It is worth noting that the colloidal compounds present in PPE, such as alkaloids, tannins, flavonoids, organic acids, and pectin may form a thin coating film on a date fruit’s surface, which may contribute to the prevention of moisture loss [35]. Our results agreed with those reported by Nair et al. [23], who found that PPE-chitosan-based film prevented capsicum fruits from losing weight during storage.

With regard to water activity, although all samples revealed a slight decrease, the water activity values remained higher than 0.900 up to the end of storage (Table 2). The decrease in water activity is mainly attributed to the decrease in the moisture content with the extended storage period. The decrease in water activity of date fruits during cold storage was also reported by Ghafoor et al. [7]. In general, there were no discernible changes in the water activity between treatments. 

All samples’ TSS concentration, a measurement of fruit ripening and maturity, dramatically rose (*p* < 0.05) (from 27.16–27.24 to 31.45–33.91%) as the storage period progressed (Table 2). The increase in the TSS level during storage could be due to the hydrolysis of polysaccharides into simple sugars [34], the conversion of some insoluble compounds to a soluble form (e.g., protopectin to pectin), and the concentration of the juice inside the fruits due to moisture loss [36]. The increase in the TSS content of the control sample during storage was higher than other treatments, and by the end of storage, the control had the highest TSS value (33.91%), while the samples treated with PPE had the lowest (31.45%). It could be suggested that the PPE may reduce the respiration rate, prevent the hydrolysis of polysaccharides, and therefore, minimize the changes in the TSS content of date fruits during storage. Similar findings have been reported by Kumar et al. [29] and Gull et al. [34] who observed that the TSS content of green bell pepper and apricot fruits increased gradually with an increase in the storage period.

The results revealed a slight decrease in pH value (from 6.00–6.28 to 4.89–5.29) with an increase in the titratable acidity (TA) (from 0.12–0.14 to 0.23–0.27% malic acid/100 g) of date samples with longer storage time (Table 2). Tabikha et al. [37] attributed the decrease in the pH of fruits and vegetables during storage to the conversion of sugars to alcohols and acids through the activity of some microorganisms. In addition, Mohammed et al. [38] reported that the pH of date fruits decreases due to natural changes that occur with the progress of maturity stages, where pH reaches the lowest value in the final stage of ripening. Similarly, Kumar et al. [29] attributed changes in pH and TA during storage to increased respiration rates and enzymatic activity. Generally, the decrease in pH and the increase in the TA of the control sample during storage were higher than other treatments. At day zero of storage, LA and LA + PPE samples showed lower pH (6.00 and 6.08, respectively) and higher TA (0.14 and 0.13%, respectively) than other samples, which could be due to the effect of the acid in the dipping solutions of these treatments. The samples treated with PPE exhibited better storage stability in terms of pH and TA than the other treatments with values of 5.28 and 0.23% after six weeks of storage, respectively. The PPE may delay the ripening process of date fruits by decreasing the respiration rate, metabolic activity, and enzymatic activity, which in turn could minimize the changes in the pH and TA during the storage period. The trends obtained in the current research are consistent with those reported by Adiamo et al. [27] and Kumar et al. [29], who observed a decrease in pH and an increase in TA in carrots and bell peppers with the passage of storage time. These authors observed that the use of natural plant extracts minimized changes in the pH and TA during the storage period.

### 3.3. Total Phenolics and Antioxidant Activity

Significant differences were observed in the total phenolic contents (TPC) between control and treated date fruits during the storage time as shown in Table 3. At day zero, the PPE and PPE + LA samples exhibited higher TPC values (12.36 and 10.47 mg GAE/g, respectively) than LA and control (8.22 and 8.24 mg GAE/g, respectively), which could be due to the presence of phenolic compounds in the PPE and PPE + LA dipping solutions, as PPE is considered to be rich in phenolic compounds. With prolonged storage, the TPC of all samples decreased gradually (from 8.22–12.36 to 3.19–5.17 mg GAE/g), which could be related to the enzymatic degradation of phenolic compounds, particularly, by PPO and POD [29]. The decrease in TPC during storage was also reported by Abdelkarim et al. [39] and Tappi et al. [40] for Barhi dates and minimally processed apples, respectively. Generally, among all samples, fruits treated with PPE effectively maintained higher TPC values compared to PPE + LA, LA, and control up to the end of storage with 5.17, 5.04, 4.12, and 3.19 mg GAE/g, respectively. PPE contains natural antioxidants which may retard oxidation reactions, delay ethylene production, and decrease enzymatic activity, which in turn may minimize the degradation of phenolic compounds of date fruits during storage. In the present study, the effects of PPE on the TPC of cold-stored Khalal Barhi dates are consistent with those indicated by Gull et al. [34] and Tappi et al. [40] for cold-stored apricot and apple fruits.

Similarly, at day zero, the PPE and PPE + LA samples exhibited higher antioxidant activity (68.26 and 58.35%, respectively) than LA and control (44.11 and 44.17%, respectively), which indicates that PPE improved the antioxidant activity of Barhi date fruits. The antioxidant activity of all samples somewhat decreased with prolonged storage (from 44.11–68.26 to 23.84–38.52%), which is consistent with the TPC data. Abdelkarim et al. [39] and Gull et al. [34] reported a reduction in antioxidant activity during the preservation of cold-stored Barhi date fruits and apricot fruits, respectively. This decrease in the antioxidant activity is mainly related to the decrease in the TPC of date samples due to the enzymatic oxidation of phenolic compounds (by PPO and POD), fruit senescence, and higher respiration rates with progressed storage time. At the end of storage, the PPE-treated samples exhibited the highest antioxidant activity (38.52%), while the control showed the lowest (23.84%). Generally, phenolic compounds are a potential source for creating antioxidant activity against oxidative damage. Accordingly, the PPE may maintain antioxidant activity through the storage period by inhibiting the oxidative destruction of bioactive compounds. In addition, PPE is a rich source of phenolic compounds (e.g., ellagitannins, gallic acid, and ellagic acid) with strong antimicrobial and antioxidant effects, consequently, it may delay the oxidation of phenolic compounds and maintain higher DPPH value during storage. 

It is important to highlight that antioxidants present in PPE may shield against several oxidation reactions caused by free radicals, prevent tissue damage, lower the danger of nutritional and functional characteristics being lost, inhibit microbial growth, and increase health benefits. Our findings agreed with those provided by Abdelkarim et al. [39] and Gull et al. [34] for cold-stored Barhi date fruits and apricot fruits, respectively; they confirmed that the natural plant extracts maintained the antioxidant activity during storage.

### 3.4. Color Measurements

One of the key characteristics that influence consumers’ decisions about food products, particularly fruits and vegetables, is color. The measurements of color attributes [L* or lightness (0 = black, 100 = white), a* (+60 = redness, −60 = greenness), and b* (+60 = yellowness, −60 = blueness)] of cold-stored Barhi date fruits treated with natural disinfection solutions are displayed in Table 4. The results revealed a slight decrease (from 54.87–55.92 to 41.68–45.68) in L* value of control and treated samples with the progression of storage time, but the control experienced a higher decrease than other samples. Similarly, Zheng et al. [41] and Gull et al. [34] reported a decrease in L* value during the cold storage of apple and apricot fruits. The decrease in L* value could be illustrated by the activity of oxidative enzymes such as polyphenol oxidase (PPO), peroxidase (POD), and catalase, which are related to the discoloration of stored fruits [7]. Moreover, the date fruits become darker (light brown) with extended storage time due to the natural ripening process (the conversion from Khalal to Rutab stage), which may be due to the degradation of native pigments and the conversion of sucrose to reducing sugars [42]. 

On contrary, the a* value of the date samples increased gradually (from 1.29–1.40 to 2.22–2.43) with the passage of storage time, showing a decrease in the greenness of the dates. Control fruit samples exhibited the greatest increase (2.43), whereas PPE treatment showed the lowest increase (2.22) during storage. Consistent with our results, a previous study conducted by Atia et al. [25] reported an increase in the a* value of Khalal Barhi dates with the progression of storage time. The increase in the a* value of cold-stored Barhi date fruits could be interpreted by the enzymatic degradation of chlorophyll pigment during the natural ripening processes. Regarding the b* value, the results revealed that all date samples exhibited a gradual decrease in b* value (from 36.25–37.09 to 23.59–25.42) during the storage period, showing a decrease in the yellowness of the dates, but the control experienced the highest decrease compared to treated samples. Our findings are consistent with the results reported by Ghafoor et al. [7] and Atia et al. [25], who observed a decrease in the b* value of Khalal Barhi dates with the extended storage. According to these scientists, the hydrolysis of carotenoid pigments, non-enzymatic Maillard browning, and the production of brown pigments was to blame for the drop in the b* value during storage.

In general, PPE-treated samples exhibited the lowest changes in the color parameters (L*, a*, b*) followed by the PPE + LA and LA samples, while control showed the greatest changes throughout the storage period. It is noteworthy that PPE is a rich source of antioxidants, which may prevent oxidative browning reactions, reduce the respiration rates and metabolic activity, delay the fruit ripening process, and retard pigment degradation, which in turn may contribute to minimizing the color changes of date fruits during storage [23,29]. The trends obtained for the effect of PPE on the color parameters in the present study are in accordance with those reported by Nair et al. [23] and Synowiec et al. [43]; they found that the natural plant extracts of PPE and sweet basil extract minimized the color changes of capsicum and apple fruits during cold storage, respectively.

### 3.5. Texture Profile 

The changes in textural parameters [hardness (kg), cohesiveness (%), and springiness (mm)] of cold-stored Barhi date samples treated with disinfection solutions are shown in Table 5. Texture profile analysis can be used to predict how a food product will behave in the mouth because it simulates the usual chewing process in the mouth. Cohesiveness describes the amount of deformation a sample undergoes before rupturing when it is bitten with molars, whereas hardness represents the food’s resistance to deformation and is defined as the required force to crush food between the molars [10]. Springiness refers to the degree and speed of a product recovery (returns its original shape or size) after compression between molars (if it is solid) or between the tongue and the palate (if it is semi-solid) [44]. 

The findings showed that the hardness, cohesiveness, and springiness of the control and treated date sample reduced as storage time increased, indicating a steady decline in fruit quality over time. The control sample showed the greatest decrease in texture properties, while the PPE-treated sample showed the least. Our results are in line with the previous studies of Alhamdan et al. [28] and Gull et al. [34], who observed a decrease in the texture properties of cold-stored Khalal Barhi dates and apricot fruits, respectively. In fact, firmness is a crucial factor that indicates the freshness and quality of fruits, particularly dates, during storage. Date softening is the loss of firmness due to the degradation of fruits’ texture resulting in lower consumer demand. It mainly occurred during storage due to the deterioration of cell structure and decomposition of the cell wall [23]. The main causes of the loss of firmness of fruits and vegetables during storage include lipid oxidation, water loss through transpiration, and pectin component degradation [29]. The most significant enzymes that reduce the mechanical strength of the cell wall throughout the ripening process and cause firmness loss are galactosidase, polygalacturonase, and pectin methylesterase [34]. 

However, the PPE treatment showed higher texture properties during storage than other samples, which could be interpreted by the ability of PPE to reduce respiration rate, slow the metabolic activity, delay the ripening process, retain the cell turgor by retarding the activity of cell wall hydrolytic enzymes, and, therefore, maintain higher texture properties. The present findings about the impact of PPE on the textural parameters of Barhi dates were consistent with previous reports of Gull et al. [34] and yang et al. [45], who observed that blueberry leaf extracts and PPE maintained better texture properties during the cold storage of blueberry and apricot fruits, respectively.

### 3.6. Sensory Evaluation

The sensory study of Barhi date fruits was done to gauge their acceptability and perception by consumers. In Table 6, the ratings of the sensory qualities (appearance, odor, taste, texture, and overall acceptability) of cold-stored Barhi date fruits with various treatments are shown. The appearance scores of all samples decreased with the advancement of storage time, which could be related to moisture loss, pigment degradation, and senescence progression [42]. However, the control sample exhibited the greatest reduction in appearance scores with prolonged storage, while PPE-treated samples revealed the least (6.40 and 7.32 at the end of storage, respectively). The PPE-treated date fruits revealed higher appearance scores up to the end of storage compared to other treatments. It is worth noting that Barhi dates treated with lactic acid (LA and PPE + LA) showed lower appearance scores than PPE, which could be related to the effect of the acid on the pigments and tissues of date fruits. 

Likewise, odor and taste scores decreased with the passage of storage time, with the greatest decrease recorded by the control and the least decrease obtained by PPE-treated samples. In addition, samples treated with LA showed lower odor (8.12) and taste (8.11) scores than PPE-treated samples (8.81 and 8.35, respectively) at day zero and during the storage period. Generally, the decrease in odor and taste scores during storage could be related to the changes accompanied by the ripening and senescence processes where the sucrose is converted to reducing sugars, the odor compounds are degraded, and secondary metabolites are formed. Seddiek et al. [19] ascribed the decline in odor and taste scores of apple samples during cold storage to respiratory metabolism and microbial growth.

Regarding the texture, it was observed that all Barhi date samples exhibited a decreasing trend in texture scores with the progression of storage time, but control exhibited the greatest decrease (6.19 after three weeks of storage), and PPE-treated samples showed the least (7.06 after six weeks of storage). Such a decrease in the texture during storage is mainly due to the enzymatic degradation of pectin substances, water loss, ripening progression, and senescence process [29,34]. It should be mentioned that the results of texture evaluation by the texture analyzer and those acquired by the sensory panel evaluation were comparable (Section 3.5.). 

Consistent with the previously discussed sensory parameters, the overall acceptability of all Barhi date samples decreased gradually (from 8.21–8.66 to 6.71–7.21) with prolonged storage. At the end of storage (3 weeks), the control showed the lowest overall acceptability score (6.71). Conversely, the samples treated with PPE exhibited better storage stability compared to other samples with an overall acceptability score of 7.21 at the end of storage (6 weeks). By reducing microbial growth, preventing enzymatic browning, reducing the degradation of pectic compounds, and reducing the respiration rate, PPE may aid in extending the shelf life of cold-stored date fruits [19]. Our findings agreed with Kumar et al.’s [29] observation that PPE preserved the sensory qualities of cold-stored bell pepper.

### 3.7. Yeasts and Molds Count

Fruits and vegetables lose quality with time and have a shorter shelf life because of microbial decomposition. Fruits typically have a low pH and high sugar content, which makes yeasts and molds more likely to cause spoil than bacterial strains [46]. Table 7 displays how sanitation procedures affected the number of yeasts and molds on Barhi date fruits when they were chilled. The treated samples had essentially no microbial growth on day 0 and after one week of storage, whereas the control had 2.13 and 2.94 log CFU/g, respectively. The results showed that the yeasts and molds count increased gradually in all samples with the progression of storage time, with the greatest increase observed in the control and the lowest increase observed in the PPE-treated sample. By the end of storage, the control sample had the highest yeast and mold count (4.21 log CFU/g), whereas the PPE-treated sample had the lowest (2.11 log CFU/g). The antimicrobial action of PPE may be responsible for the reduced yeast and mold count in fruits treated with it [34]. The shelf lives of cold-stored Barhi date fruits were 3 weeks for the control, but it reached 4, 5, and 6 weeks for the LA, LA + PPE, and PPE-treated samples, respectively. However, the yeasts and molds count of all samples in the current study remained below the maximum permissible limit (6.0 log CFU/g) prescribed for fruit quality throughout the storage period [19]. Our findings are in line with those reported by Nair et al. [23] and Gull et al. [34], who observed that PPE reduced the yeasts and molds counts during the cold storage of capsicum and apricot fruits, respectively.

## 4. Conclusions

In contrast to the control sample, the current investigation found that dipping treatments with natural preservatives increased the shelf life of Khalal Barhi date fruits. All treatments increased soluble solids, prevented moisture, color, and texture loss during storage, maintained the sensory characteristics of Barhi date fruits, and inhibited fungal growth. Moreover, the treatments improved phenolics content and antioxidant activity and minimized their decrease with the advancement of storage. Among the treatments, PPE-treated samples showed the best storage stability in terms of all parameters evaluated. It can be concluded that PPE dipping treatment may be beneficial to extend the shelf life and preserve the quality of Barhi date fruits. Further investigations into processing conditions and applications in the food industry are needed.

## Figures and Tables

**Table 1:** HPLC analysis of polyphenolic compounds in pomegranate peel extract.

Polyphenolic Compound	Conc. (mg/g) *
Gallic acid	12.50 ± 0.36
Chlorogenic acid	1.19 ± 0.09
Catechin	4.10 ± 0.17
Methyl gallate	0.21 ± 0.03
Caffeic acid	0.05 ± 0.01
Syringic acid	0.31 ± 0.04
Pyro catechol	ND
Rutin	0.07 ± 0.01
Ellagic acid	35.12 ± 0.94
Coumaric acid	ND
Vanillin	0.07 ± 0.01
Ferulic acid	0.53 ± 0.07
Naringenin	1.29 ± 0.09
Daidzein	ND
Quercetin	ND
Cinnamic acid	ND
Apigenin	0.18 ± 0.02
Kaempferol	ND
Hesperetin	ND

* The results are presented as the means ± standard deviation; ND not detected.

**Table 2:** Changes in moisture content, water activity, total soluble solids, pH, and titratable acidity of Barhi dates treated with natural disinfectants during cold storage (4 ± 1 °C).

Treatments	Storage Period (Week)						
	0	1	2	3	4	5	6
	Moisture content (%)						
Control	68.41 ± 1.25 ^Aa^	67.05 ± 0.92 ^Aa^	65.24 ± 1.75 ^Ab^	63.11 ± 0.72 ^Bc^	-	-	-
LA	68.36 ± 1.43 ^Aa^	67.48 ± 1.73 ^Aab^	65.71 ± 1.33 ^Abc^	64.28 ± 1.40 ^ABcd^	62.39 ± 1.31 ^Ad^	-	-
PPE	68.43 ± 0.98 ^Aa^	68.03 ± 1.17 ^Aa^	67.52 ± 0.92 ^Aab^	65.85 ± 0.83 ^Abc^	64.71 ± 1.53 ^Acd^	63.17 ± 1.22 ^Ade^	62.27 ± 1.38 ^Ae^
LA + PPE	68.37 ± 0.80 ^Aa^	67.72 ± 1.45 ^Aa^	66.94 ± 0.81 ^Aab^	65.27 ± 0.89 ^Abc^	64.08 ± 1.27 ^Ac^	62.13 ± 1.34 ^Ad^	-
	Water activity						
Control	0.938 ± 0.03 ^Aa^	0.929 ± 0.04 ^Aa^	0.919 ± 0.01 ^Aa^	0.913 ± 0.07 ^Aa^	-	-	-
LA	0.936 ± 0.01 ^Aa^	0.930 ± 0.02 ^Aa^	0.922 ± 0.07 ^Aa^	0.918 ± 0.04 ^Aa^	0.914 ± 0.04 ^Aa^	-	-
PPE	0.939 ± 0.02 ^Aa^	0.934 ± 0.06 ^Aa^	0.926 ± 0.02 ^Aa^	0.922 ± 0.07 ^Aa^	0.919 ± 0.05 ^Aa^	0.918 ± 0.04 ^Aa^	0.916 ± 0.08 ^Aa^
LA + PPE	0.938 ± 0.04 ^Aa^	0.932 ± 0.02 ^Aa^	0.925 ± 0.06 ^Aa^	0.920 ± 0.03 ^Aa^	0.917 ± 0.03 ^Aa^	0.915 ± 0.07 ^Aa^	-
	Total soluble solids (%)						
Control	27.16 ± 0.52 ^Ad^	29.41 ± 0.84 ^Ac^	32.81 ± 0.61 ^Ab^	33.91 ± 0.80 ^Aa^	-	-	-
LA	27.18 ± 0.45 ^Ad^	28.85 ± 0.71 ^Ac^	30.16 ± 0.95 ^Bb^	31.47 ± 0.33 ^Ba^	32.05 ± 0.54 ^Aa^	-	-
PPE	27.24 ± 0.71 ^Ae^	28.30 ± 0.43 ^Ad^	29.23 ± 0.52 ^Bcd^	30.05 ± 0.74 ^Cbc^	30.84 ± 0.43 ^Bab^	31.11 ± 0.42 ^Aa^	31.45 ± 0.37 ^Aa^
LA + PPE	27.20 ± 0.90 ^Ae^	28.56 ± 0.57 ^Ad^	29.54 ± 0.43 ^Bcd^	30.37 ± 0.44 ^BCbc^	31.09 ± 0.72 ^Bab^	31.63 ± 0.65 ^Aa^	-
	pH						
Control	6.28 ± 0.21 ^Aa^	6.01 ± 0.62 ^Aab^	5.63 ± 0.30 ^Ab^	4.89 ± 0.51 ^Ac^	-	-	-
LA	6.00 ± 0.56 ^Aa^	5.92 ± 0.09 ^Aab^	5.83 ± 0.27 ^Aab^	5.60 ± 0.72 ^Aab^	5.26 ± 0.19 ^Bb^	-	-
PPE	6.19 ± 0.32 ^Aa^	6.15 ± 0.43 ^Aa^	6.04 ± 0.16 ^Aa^	5.91 ± 0.65 ^Aab^	5.79 ± 0.41 ^Aab^	5.51 ± 0.26 ^Aab^	5.28 ± 0.17 ^Ab^
LA + PPE	6.08 ± 0.18 ^Aa^	6.03 ± 0.22 ^Aa^	5.92 ± 0.51 ^Aab^	5.75 ± 0.38 ^Aabc^	5.42 ± 0.17 ^ABbc^	5.29 ± 0.31 ^Ac^	-
	Titratable acidity (% malic acid/100 g)						
Control	0.12 ± 0.03 ^Ad^	0.16 ± 0.02 ^Ac^	0.21 ± 0.04 ^Ab^	0.27 ± 0.01 ^Aa^	-	-	-
LA	0.14 ± 0.07 ^Ab^	0.16 ± 0.05 ^Aab^	0.19 ± 0.05 ^Aab^	0.21 ± 0.04 ^ABab^	0.24 ± 0.07 ^Aa^	-	-
PPE	0.12 ± 0.05 ^Ac^	0.13 ± 0.04 ^Ac^	0.15 ± 0.01 ^Abc^	0.18 ± 0.05 ^Babc^	0.20 ± 0.04 ^Aab^	0.21 ± 0.02 ^Aab^	0.23 ± 0.03 ^Aa^
LA + PPE	0.13 ± 0.03 ^Ad^	0.14 ± 0.03 ^Acd^	0.17 ± 0.02 ^Abcd^	0.20 ± 0.03 ^ABabc^	0.21 ± 0.06 ^Aab^	0.24 ± 0.05 ^Aa^	-

The results are presented as the means ± standard deviation; *n* = 3. Means followed by different superscript letters within each column (upper case) or row (lower case) are significantly different (*p* < 0.05). LA, lactic acid; PPE, pomegranate peel extract; -, not determined due to sensorial rejection or spoilage.

**Table 3:** Changes in total phenolic content and antioxidant activity (DPPH scavenging) of Barhi dates treated with natural disinfectants during cold storage (4 ± 1 °C).

Treatments	Storage Period (Week)						
	0	1	2	3	4	5	6
	Total phenolic content (mg GAE/g)						
Control	8.24 ± 0.21 ^Ca^	6.19 ± 0.40 ^Cb^	4.46 ± 0.35 ^Dc^	3.19 ± 0.61 ^Dd^	-	-	-
LA	8.22 ± 0.45 ^Ca^	6.67 ± 0.18 ^Cb^	5.71 ± 0.19 ^Cc^	4.80 ± 0.52 ^Cd^	4.12 ± 0.42 ^Ce^	-	-
PPE	12.36 ± 0.53 ^Aa^	11.08 ± 0.69 ^Ab^	9.48 ± 0.34 ^Ac^	8.33 ± 0.37 ^Ad^	7.04 ± 0.35 ^Ae^	6.22 ± 0.27 ^Af^	5.17 ± 0.42 ^Ag^
LA + PPE	10.47 ± 0.18 ^Ba^	9.13 ± 0.43 ^Bb^	8.21 ± 0.60 ^Bc^	6.58 ± 0.35 ^Bd^	5.71 ± 0.34 ^Be^	5.04 ± 0.18 ^Bf^	-
	DPPH scavenging (%)						
Control	44.17 ± 0.98 ^Ca^	36.22 ± 0.78 ^Cb^	27.45 ± 0.91 ^Dc^	23.84 ± 0.75 ^Dd^	-	-	-
LA	44.11 ± 1.09 ^Ca^	37.26 ± 1.25 ^Cb^	31.45 ± 1.16 ^Cc^	29.14 ± 0.86 ^Cd^	24.52 ± 1.14 ^Ce^	-	-
PPE	68.26 ± 0.87 ^Aa^	63.74 ± 1.12 ^Ab^	54.19 ± 1.27 ^Ac^	49.77 ± 1.37 ^Ad^	44.89 ± 1.27 ^Ae^	40.24 ± 1.09 ^Af^	38.52 ± 0.93 ^Af^
LA + PPE	58.35 ± 1.36 ^Ba^	52.44 ± 1.32 ^Bb^	49.82 ± 1.41 ^Bc^	41.63 ± 0.94 ^Bd^	39.13 ± 1.44 ^Be^	33.40 ± 1.23 ^Bf^	-

The results are presented as the means ± standard deviation; *n* = 3. Means followed by different superscript letters within each column (upper case) or row (lower case) are significantly different (*p* < 0.05). LA, lactic acid; PPE, pomegranate peel extract; -, not determined due to sensorial rejection or spoilage.

**Table 4:** Changes in color parameters of Barhi dates treated with natural disinfectants during cold storage (4 ± 1 °C).

Treatments	Storage Period (Week)						
	0	1	2	3	4	5	6
	L*						
Control	55.32 ± 1.32 ^Aa^	50.26 ± 0.87 ^Bb^	44.53 ± 1.22 ^Bc^	41.68 ± 0.84 ^Bd^	-	-	-
LA	55.92 ± 0.81 ^Aa^	51.75 ± 1.14 ^ABb^	49.24 ± 1.17 ^Ac^	48.19 ± 0.97 ^Ac^	43.75 ± 1.20 ^Cd^	-	-
PPE	54.87 ± 1.13 ^Aa^	52.16 ± 0.78 ^Ab^	50.46 ± 0.87 ^Abc^	49.17 ± 1.26 ^Acd^	48.36 ± 0.81 ^Ad^	47.65 ± 1.14 ^Ad^	45.68 ± 1.32 ^Ae^
LA + PPE	55.76 ± 1.37 ^Aa^	51.84 ± 0.82 ^ABb^	49.80 ± 0.91 ^Ac^	48.92 ± 1.24 ^Ac^	46.15 ± 0.78 ^Bd^	45.50 ± 0.93 ^Bd^	-
	a*						
Control	1.29 ± 0.04 ^Bd^	1.54 ± 0.07 ^Ac^	1.85 ± 0.08 ^Ab^	2.43 ± 0.03 ^Aa^	-	-	-
LA	1.36 ± 0.05 ^ABd^	1.49 ± 0.03 ^Ac^	1.75 ± 0.08 ^ABb^	1.82 ± 0.09 ^Bb^	2.30 ± 0.05 ^Aa^	-	-
PPE	1.40 ± 0.07 ^Ae^	1.46 ± 0.09 ^Ae^	1.61 ± 0.08 ^Bd^	1.68 ± 0.05 ^Cd^	1.91 ± 0.06 ^Cc^	2.10 ± 0.05 ^Bb^	2.22 ± 0.06 ^Aa^
LA + PPE	1.34 ± 0.05 ^ABe^	1.48 ± 0.07 ^Ad^	1.64 ± 0.09 ^Bc^	1.74 ± 0.07 ^BCc^	2.05 ± 0.03 ^Bb^	2.27 ± 0.06 ^Aa^	-
	b*						
Control	36.25 ± 0.92 ^Aa^	32.11 ± 1.09 ^Bb^	26.47 ± 0.88 ^Bc^	23.59 ± 0.76 ^Cd^	-	-	-
LA	36.68 ± 1.23 ^Aa^	33.58 ± 1.40 ^ABb^	30.05 ± 0.92 ^Ac^	26.19 ± 0.75 ^Bd^	25.42 ± 0.84 ^Ad^	-	-
PPE	36.43 ± 0.85 ^Aa^	34.66 ± 0.76 ^Ab^	31.65 ± 1.14 ^Ac^	29.44 ± 1.20 ^Ad^	26.19 ± 1.33 ^Ae^	24.75 ± 0.76 ^Aef^	24.11 ± 0.64 ^Af^
LA + PPE	37.09 ± 0.79 ^Aa^	34.15 ± 1.17 ^Ab^	30.94 ± 0.84 ^Ac^	28.43 ± 0.91 ^Ad^	26.00 ± 0.79 ^Ae^	23.86 ± 1.04 ^Af^	-

The results are presented as the means ± standard deviation; *n* = 3. Means followed by different superscript letters within each column (upper case) or row (lower case) are significantly different (*p* < 0.05). L* (0 = darkness, 100 = lightness); a* (−60 = greenness, +60 = redness); b* (−60 = blueness, +60 = yellowness); LA, lactic acid; PPE, pomegranate peel extract; -, not determined due to sensorial rejection or spoilage.

**Table 5:** Changes in texture profile of Barhi dates treated with natural disinfectants during cold storage (4 ± 1 °C).

Treatments	Storage Period (Week)						
	0	1	2	3	4	5	6
	Hardness (kg)						
Control	1055 ± 7 ^Aa^	732 ± 4 ^Db^	237 ± 5 ^Dc^	83 ± 3 ^Dd^	-	-	-
LA	1041 ± 5 ^Ba^	745 ± 7 ^Cb^	315 ± 8 ^Cc^	143 ± 3 ^Cd^	79 ± 2 ^Ce^	-	-
PPE	1064 ± 9 ^Aa^	821 ± 5 ^Ab^	406 ± 4 ^Ac^	250 ± 5 ^Ad^	176 ± 3 ^Ae^	122 ± 4 ^Af^	85 ± 3 ^Ag^
LA + PPE	1058 ± 8 ^Aa^	779 ± 6 ^Bb^	371 ± 7 ^Bc^	184 ± 3 ^Bd^	113 ± 3 ^Bde^	81 ± 2 ^Bde^	-
	Cohesiveness						
Control	0.81 ± 0.03 ^Aa^	0.77 ± 0.05 ^Aab^	0.75 ± 0.02 ^Aab^	0.72 ± 0.08 ^Ab^	-	-	-
LA	0.80 ± 0.05 ^Aa^	0.79 ± 0.02 ^Aa^	0.78 ± 0.06 ^Aa^	0.76 ± 0.05 ^Aa^	0.73 ± 0.07 ^Aa^	-	-
PPE	0.87 ± 0.04 ^Aa^	0.85 ± 0.03 ^Aa^	0.82 ± 0.04 ^Aab^	0.81 ± 0.03 ^Aab^	0.79 ± 0.06 ^Aab^	0.78 ± 0.04 ^Aab^	0.75 ± 0.06 ^Ab^
LA + PPE	0.85 ± 0.07 ^Aa^	0.83 ± 0.08 ^Aa^	0.80 ± 0.09 ^Aa^	0.79 ± 0.03 ^Aa^	0.76 ± 0.02 ^Aa^	0.74 ± 0.08 ^Aa^	-
	Springiness (mm)						
Control	0.90 ± 0.07 ^Aa^	0.86 ± 0.09 ^Aab^	0.81 ± 0.03 ^Ab^	0.78 ± 0.05 ^Ab^	-	-	-
LA	0.91 ± 0.03 ^Aa^	0.89 ± 0.05 ^Aa^	0.87 ± 0.07 ^Aa^	0.83 ± 0.06 ^Aab^	0.78 ± 0.03 ^Ab^	-	-
PPE	0.94 ± 0.05 ^Aa^	0.92 ± 0.08 ^Aab^	0.91 ± 0.04 ^Aabc^	0.88 ± 0.03 ^Aabcd^	0.84 ± 0.02 ^Abcd^	0.82 ± 0.06 ^Acd^	0.81 ± 0.03 ^Ad^
LA + PPE	0.92 ± 0.06 ^Aa^	0.91 ± 0.07 ^Aab^	0.89 ± 0.09 ^Aab^	0.86 ± 0.08 ^Aab^	0.83 ± 0.07 ^Aab^	0.79 ± 0.04 ^Ab^	-

The results are presented as the means ± standard deviation; *n* = 3. Means followed by different superscript letters within each column (upper case) or row (lower case) are significantly different (*p* < 0.05). LA, lactic acid; PPE, pomegranate peel extract; -, not determined due to sensorial rejection or spoilage.

**Table 6:** Changes in sensory properties of Barhi dates treated with natural disinfectants during cold storage (4 ± 1 °C).

Treatments	Storage Period (Week)						
	0	1	2	3	4	5	6
	Appearance						
Control	8.13 ± 0.61 ^Aa^	7.58 ± 0.70 ^Aab^	7.12 ± 0.62 ^Bbc^	6.40 ± 0.86 ^Bc^	-	-	-
LA	8.11 ± 0.45 ^Aa^	7.90 ± 0.95 ^Aa^	7.52 ± 0.36 ^ABab^	6.84 ± 0.58 ^ABbc^	6.43 ± 0.37 ^Cc^	-	-
PPE	8.84 ± 0.12 ^Aa^	8.67 ± 0.33 ^Aa^	8.35 ± 0.41 ^Aab^	8.13 ± 0.67 ^Aabc^	7.94 ± 0.48 ^Aabc^	7.69 ± 0.62 ^Abc^	7.32 ± 0.53 ^Ac^
LA + PPE	8.44 ± 0.37 ^Aa^	8.23 ± 0.74 ^Aab^	8.16 ± 0.48 ^Aab^	7.87 ± 0.59 ^Aab^	7.26 ± 0.29 ^Bbc^	6.51 ± 0.71 ^Bc^	-
	Odor						
Control	8.25 ± 0.29 ^Aa^	8.03 ± 0.37 ^Aab^	7.76 ± 0.48 ^Ab^	7.11 ± 0.17 ^Bc^	-	-	-
LA	8.12 ± 0.57 ^Aa^	8.01 ± 0.48 ^Aab^	7.80 ± 0.54 ^Aab^	7.35 ± 0.79 ^ABab^	7.16 ± 0.36 ^Bb^	-	-
PPE	8.81 ± 0.18 ^Aa^	8.60 ± 0.37 ^Aab^	8.53 ± 0.32 ^Aab^	8.42 ± 0.52 ^Aab^	8.26 ± 0.62 ^Aab^	7.82 ± 0.52 ^Abc^	7.35 ± 0.48 ^Ac^
LA + PPE	8.31 ± 0.53 ^Aa^	8.25 ± 0.51 ^Aa^	8.14 ± 0.29 ^Aab^	7.68 ± 0.78 ^ABab^	7.45 ± 0.39 ^Bab^	7.19 ± 0.80 ^Ab^	-
	Taste						
Control	8.43 ± 0.29 ^Aa^	8.11 ± 0.18 ^Aab^	7.63 ± 0.90 ^Abc^	7.10 ± 0.48 ^Ac^	-	-	-
LA	8.11 ± 0.63 ^Aa^	8.02 ± 0.55 ^Aa^	7.82 ± 0.63 ^Aa^	7.70 ± 0.70 ^Aa^	7.12 ± 0.53 ^Aa^	-	-
PPE	8.35 ± 0.48 ^Aa^	8.23 ± 0.71 ^Aa^	8.14 ± 0.58 ^Aa^	8.07 ± 0.81 ^Aa^	7.92 ± 0.65 ^Aa^	7.75 ± 0.44 ^Aa^	7.16 ± 0.53 ^Aa^
LA + PPE	8.23 ± 0.61 ^Aa^	8.14 ± 0.82 ^Aa^	8.05 ± 0.29 ^Aa^	7.93 ± 0.92 ^Aa^	7.74 ± 0.38 ^Aa^	7.20 ± 0.61 ^Aa^	-
	Texture						
Control	8.60 ± 0.29 ^Aa^	8.04 ± 0.80 ^Aab^	7.56 ± 0.90 ^Ab^	6.19 ± 0.82 ^Bc^	-	-	-
LA	8.51 ± 0.40 ^Aa^	8.33 ± 0.19 ^Aa^	8.12 ± 0.45 ^Aa^	7.81 ± 0.90 ^Aa^	7.05 ± 0.30 ^Ab^	-	-
PPE	8.64 ± 0.31 ^Aa^	8.51 ± 0.48 ^Aa^	8.34 ± 0.62 ^Aa^	8.09 ± 0.63 ^Aab^	7.74 ± 0.57 ^Aabc^	7.30 ± 0.46 ^Abc^	7.06 ± 0.52 ^Ac^
LA + PPE	8.62 ± 0.23 ^Aa^	8.40 ± 0.55 ^Aa^	8.29 ± 0.62 ^Aab^	8.00 ± 0.19 ^Aab^	7.52 ± 0.62 ^Abc^	7.05 ± 0.38 ^Ac^	-
	Overall acceptability						
Control	8.35 ± 0.52 ^Aa^	7.94 ± 0.62 ^Aab^	7.52 ± 0.29 ^Ab^	6.71 ± 0.51 ^Bc^	-	-	-
LA	8.21 ± 0.60 ^Aa^	8.07 ± 0.73 ^Aa^	7.82 ± 0.42 ^Aa^	7.43 ± 0.39 ^ABab^	6.89 ± 0.45 ^Bb^	-	-
PPE	8.66 ± 0.43 ^Aa^	8.50 ± 0.80 ^Aab^	8.34 ± 0.18 ^Aab^	8.18 ± 0.80 ^Aab^	7.97 ± 0.72 ^Aabc^	7.64 ± 0.53 ^Abc^	7.21 ± 0.37 ^Ac^
LA + PPE	8.40 ± 0.39 ^Aa^	8.28 ± 0.61 ^Aa^	8.16 ± 0.90 ^Aa^	7.87 ± 0.18 ^ABab^	7.49 ± 0.81 ^ABab^	6.99 ± 0.72 ^Ab^	-

The results are presented as the means ± standard deviation; *n* = 3. Means followed by different superscript letters within each column (upper case) or row (lower case) are significantly different (*p* < 0.05). LA, lactic acid; PPE, pomegranate peel extract; -, not determined due to sensorial rejection or spoilage.

**Table 7:** Changes in yeasts and molds count (log CFU/g) of Barhi dates treated with natural disinfectants during cold storage (4 ± 1 °C).

Treatments	Storage Period (Week)						
	0	1	2	3	4	5	6
Control	2.13 ± 0.11 ^Ad^	2.94 ± 0.19 ^Ac^	3.44 ± 0.21 ^Ab^	4.21 ± 0.19 ^Aa^	-	-	-
LA	n.d	n.d	0.54 ± 0.10 ^Bc^	1.28 ± 0.14 ^Bb^	2.72 ± 0.14 ^Aa^	-	-
PPE	n.d	n.d	0.59 ± 0.14 ^Bd^	1.13 ± 0.18 ^Bc^	1.55 ± 0.17 ^Bb^	1.93 ± 0.17 ^Ba^	2.11 ± 0.16 ^Aa^
LA + PPE	n.d	n.d	0.35 ± 0.09 ^Bd^	1.00 ± 0.12 ^Bc^	1.33 ± 0.15 ^Bb^	2.43 ± 0.18 ^Aa^	-

The results are presented as the means ± standard deviation; *n* = 3. Means followed by different superscript letters within each column (upper case) or row (lower case) are significantly different (*p* < 0.05). LA, lactic acid; PPE, pomegranate peel extract; n.d, not detected; -, not determined due to sensorial rejection or spoilage.

## Data Availability

The data are available upon request.

## Supplementary Materials

The following supporting information can be downloaded at: https://www.mdpi.com/article/10.3390/foods12061160/s1, Figure S1: HPLC chromatogram of pomegranate peel extract; Table S1: HPLC analysis of polyphenolic compounds in the standards.
