# Supplementary material for: Effects of Pomegranate Peel Extract and/or Lactic Acid as Natural Preservatives on Physicochemical, Microbiological Properties, Antioxidant Activity, and Storage Stability of Khalal Barhi Date Fruits
Source: Foods. 2023 Mar 9;12(6):1160. doi: 10.3390/foods12061160 (PMC10048100; doi:10.3390/foods12061160)
Supplement: Supplementary file 1 [file foods-12-01160-s001.zip › foods-2233688-supplementary.pdf]

Supplementary file Manuscript ID: foods-2233688

Table S1. HPLC analysis of polyphenolic compounds in the standards.

**Polyphenolic compound**	Conc. (µg/ml)
Gallic acid	15
Chlorogenic acid	50
Catechin	75
Methyl gallate	15
Caffeic acid	18
Syringic acid	17.2
Pyro catechol	40
Rutin	26
Ellagic acid	120
Coumaric acid	20
Vanillin	12.9
Ferulic acid	20
Naringenin	30
Daidzein	35
Quercetin	40
Cinnamic acid	10
Apigenin	50
Kaempferol	20
Hesperetin	20

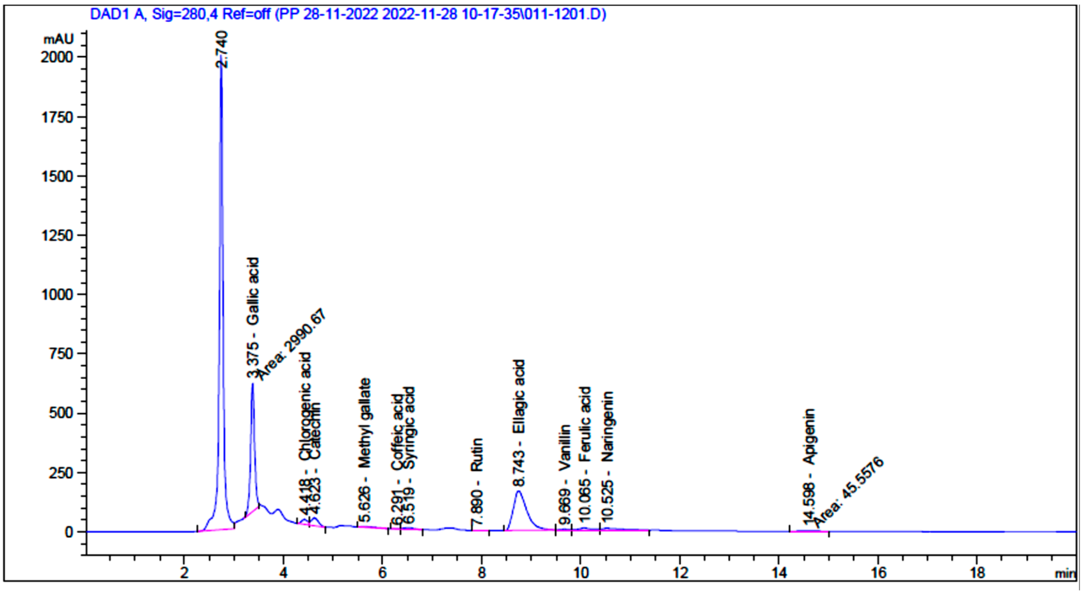

Figure S1. HPLC chromatogram of pomegranate peel extract.
